# Theoretical study of a highly fault-tolerant and scalable adaptive radiative cooler

**DOI:** 10.1515/nanoph-2023-0739

**Published:** 2024-02-12

**Authors:** Bin Li, Jiaqi Hu, Changhao Chen, Hengren Hu, Yetao Zhong, Ruichen Song, Boyu Cao, Yunqi Peng, Xusheng Xia, Kai Chen, Zhilin Xia

**Affiliations:** Wuhan University of Technology, State Key Laboratory of Silicate Materials for Architectures, Wuhan, China; Wuhan Zhongyuan Huadian Science and Technology Co., Ltd., Wuhan, China

**Keywords:** passive radiative cooling, vanadium dioxide, adaptive, thermal emission, thickness optimization

## Abstract

Conventional static radiative coolers have an unadjustable cooling capacity, which often results in overcooling in low temperature environment. Therefore, there is a great need for an adaptive dynamic radiative cooler. However, such adaptive coolers usually require complex preparation processes. This paper proposes an adaptive radiative cooler based on a Fabry–Perot resonant cavity. By optimizing the structural parameters of the radiative cooler, this adaptive radiative cooler achieves a modulation rate of 0.909 in the atmospheric window band. The net radiative cooling performance difference between low and high temperatures is nearly eight times. Meanwhile, the device is easily prepared, has a high tolerance, and can effectively prevent W–VO_2_ oxidation. This study provides new insights into adaptive radiative cooling with potential for large-scale applications.

## 1 Introduction

Passive radiative cooling uses the “transparent window” of the atmosphere to release the heat of an object on Earth into space through thermal radiative. At the same time, only a trace amount of thermal radiative from space is absorbed, resulting in passive radiative cooling of the object [1]. Passive radiative cooling techniques have been studied extensively over the past decades but mainly focused on nighttime radiative cooling [2] until 2014, when all-day radiative cooling was realized [3]. Since then, materials have been developed that enable all-day radiative cooling. These include polymer films [4], [5], metamaterials [6], multilayer dielectric films [7], cellulose [8], and nanocoatings [9]. The maximum radiative cooling capacity during the day has reached about 40 K below the ambient temperature, showing great potential for application [10]. Meanwhile, passive radiative cooling has emerged as a new cooling technology. It can reduce the object’s temperature to below the ambient temperature under zero energy consumption and zero emission, which has incomparable advantages in energy saving, emission reduction, and environmental protection [11].

Although significant advancements have been made in radiative cooling technology, most existing radiative cooling systems are considered “static” since the thermal emissivity of the system remains fixed to high infrared emissivity throughout [12]. However, cooling may only sometimes be necessary in some cases. For instance, buildings require cooling during summer and heating during winter. If the passive radiative cooling material used still has a cooling effect in winter, it fails to meet the need for temperature control and is hard to use broadly. Radiant materials do not possess modifiable cooling capability; the energy conserved at high temperatures will be expended at low temperatures [13]. Consequently, there is a pressing requirement for an “adaptive” radiative cooling system. It can achieve radiative cooling in high ambient temperatures and reduce or even switch it off in low temperatures.

Recently, some researchers have designed and fabricated adaptive radiative coolers by simulation or experimental methods. Xia et al. [14] designed a mechanical structure based on a memory metal, which can turn on and off the radiative cooling according to the temperature. Wang et al. [15] prepared an adaptive coating based on a reversible thermochromic capsule, whose temperature difference between the heating and cooling states reached 9.5 °C. Chen et al. [16] designed a bright window with thermochromic hydrogel, which achieved a solar modulation rate of 88.84 %. Tang et al. [17] designed a flexible coating structure by patterned etching on the VO_2_ surface, achieving a modulation rate of 0.7 in the atmospheric window band. Gu et al. [18] designed an adaptive coating consisting of VO_2_/HfO_2_/Al composed of an F–P resonant cavity structure for an innovative IR radiative modulator, and the tunability of the emissivity reached 0.51. Long et al. [13] designed and fabricated a scalable smart window based on VO_2_ that provides different emissivities to regulate radiative cooling at high and low temperatures, providing a good solution for energy-efficient buildings. Although these researchers realized adaptive radiative cooling, there are still some problems. For example, the mechanical switch structure is complicated and relatively bulky; the microcapsules are prone to leakage and failure; and the hydrogel is cumbersome to prepare and difficult to apply on a large scale. VO_2_, as a typical phase-change material, can change its infrared optical properties through thermal induction. Using VO_2_ as a thermal emission switch does not require additional power input or feedback control, significantly reducing the system’s complexity [19], [20]. However, the VO_2_ preparation process is cumbersome and costly, and the preparation is less fault-tolerant [21]. Meanwhile, VO_2_ is very easy to oxidize by air, thus losing the tunability of IR emission [22]. How to solve the above problems is still a significant challenge.

In this work, we designed an adaptive radiative cooling system based on W–VO_2_. The system consists of a solar reflector at the top and a radiative cooler at the bottom with large IR emission tunability. The bottom radiative cooler consists of POE + W–VO_2_/POE/Ag (POE is ethylene–octene copolymer [Poly(ethylene-1-octene)], W–VO_2_ is W-doped vanadium dioxide), where the thickness of the POE + W–VO_2_ hybrid layer can reach the micrometer level. The preparation is highly tolerant and can be prepared by a simple spin-coating or dip-lifting process. Meanwhile, the hybrid layer can protect W–VO_2_ well. Preventing it from oxidizing and losing its regulating ability has potential application value by optimizing the thicknesses of the hybrid and POE layers and the ratio of W–VO_2_ doping in the bottom radiative cooler. The adaptive radiative cooler achieves a modulation rate 0.909 in the atmospheric window band, with a nearly 8-fold difference in net radiative cooling power at low and high temperatures. The adaptive radiative cooler has excellent dynamic thermal management capability, simple process, and low cost and has a broad application prospect.

## 2 Conceptual and structural design

### 2.1 Adaptive radiative cooling concept

**Figure 1:**
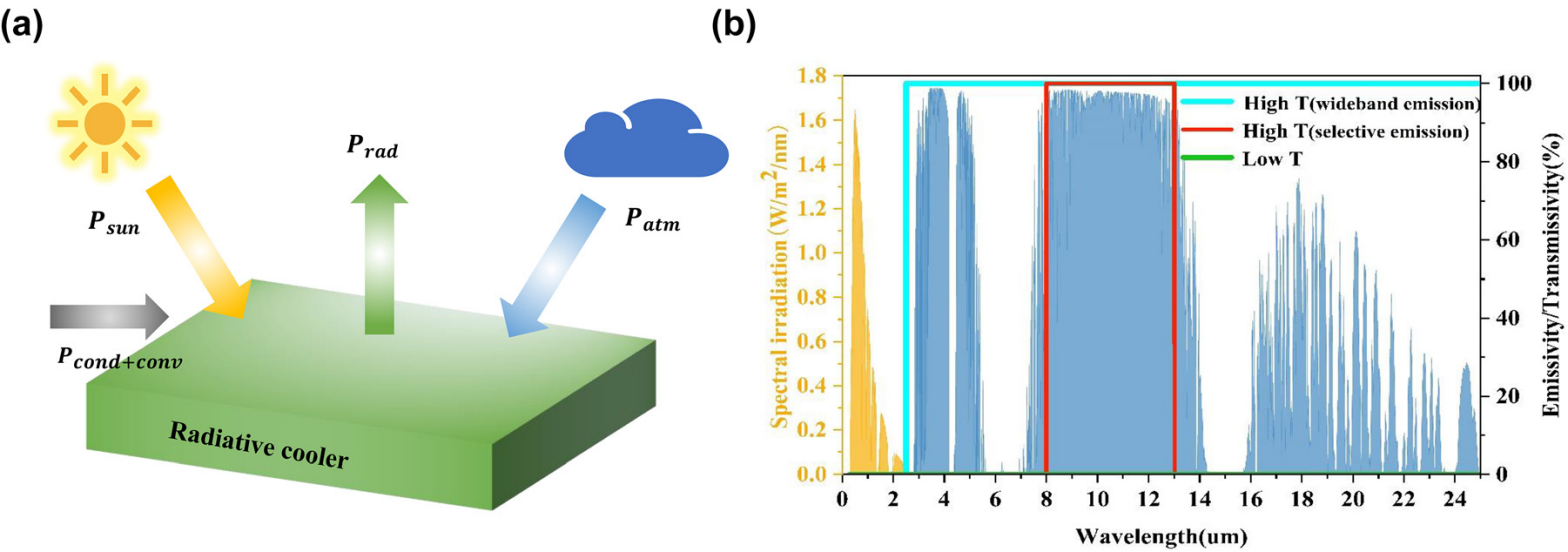
Schematic diagram of the radiative cooling energy flow and spectrogram of an ideal adaptive radiative cooler. (a) Energy composition of the radiative cooler exposed to the atmosphere; (b) spectral diagram of an ideal adaptive radiative cooler, with the green line indicating the emissivity at low temperatures and the red and blue lines indicating the ideal emissivity for both emitters at high temperatures. The yellow shading indicates the standard AM1.5 solar radiation spectral distribution, and the blue shading indicates the atmospheric transmission spectral distribution.

The principle of passive radiative cooling is shown in Figure 1(a). In order to realize adaptive radiative cooling, the system needs to have different spectral characteristics at high and low temperatures [23]. An ideal adaptive radiative cooling system should have a spectral signature as shown in Figure 1(b): when the temperature is low, the system absorbs 0 in the 8–13 μm band. At this time, the radiative cooling system is off, thus avoiding unwanted cooling to retain the object’s heat [24]. At higher temperatures, the system should have an absorptivity of 0 in the 0.3–2.5 μm band to minimize the absorption of thermal radiation from the sun. In other bands, there are two design options: broadband emission and selective emission. The differences between the two designs have been the subject of many previous studies [25], [26] and will not be repeated in this paper. Both require high emissivity in the atmospheric window band when the radiative cooling system is switched on, thus effectively emitting heat into outer space.

**Figure 2:**
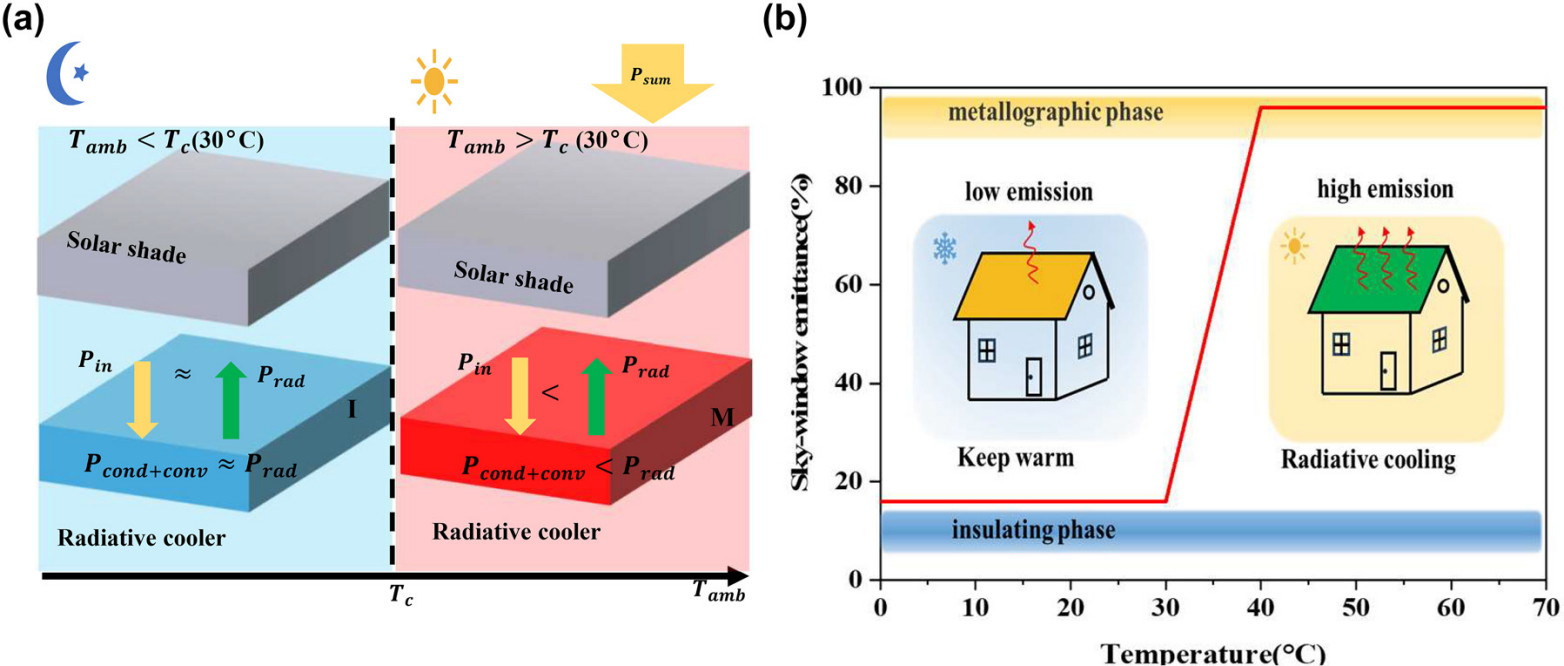
Schematic diagram of the adaptive radiative cooler and thermal management. (a) The schematic diagram of the adaptive radiative cooler concept switches on radiative cooling when the temperature is above the critical temperature *T*_*c*_ and switches off radiative cooling when the temperature is below the critical temperature. (b) Schematic diagram of the atmospheric window emissivity and thermal management of the adaptive radiative cooler.

Figure 2 is a schematic diagram of the principle of the adaptive radiative cooling system we designed. The system has a solar reflector at the top and a radiative cooler at the bottom. As shown in Figure 2(a), at night (or in cold weather), the bottom radiative cooler is switched to low emissivity, resulting in *P*_*rad*_ ≈ *P*_*in*_, and the radiative cooling is closed. During the daytime (or hot weather), the high temperature causes the bottom radiant cooler to switch to a high emissivity state. At this time, the emission power of the radiative cooler is greater than the input power through the solar reflector, that is, *P*_*rad*_ > *P*_*in*_, and the radiant cooling is in an open state. Figure 2(b) is the dynamic thermal management diagram [17] of the adaptive radiation cooling system, which can realize the dynamic thermal management [27] according to the temperature change.

### 2.2 Design and principle of Fabry–Perot resonant cavity

**Figure 3:**
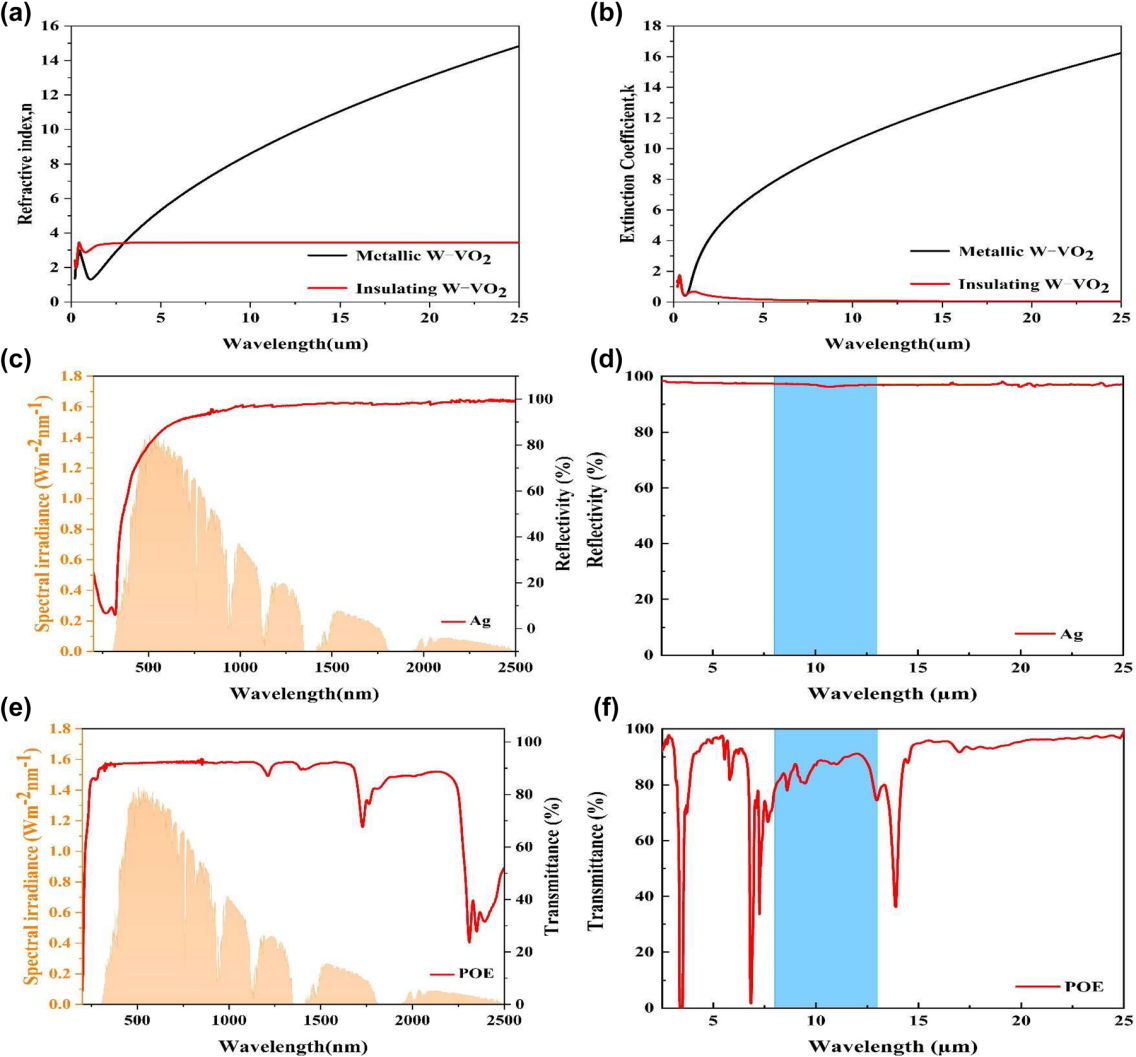
Optical properties of W–VO_2_, Ag, and POE. (a) Refractive index *n* of W–VO_2_ in the insulating and metallic phases; (b) extinction coefficient *k* of W–VO_2_ in the insulating and metallic phases. (c) Reflectance spectra of Ag in the UV–visible near-infrared wavelength band; (d) reflectance spectra of Ag in the mid- and far-infrared wavelength bands. (e) Transmittance spectra of POE in the UV–visible near-infrared band; (f) transmittance spectra of POE in the mid- and far-infrared band.

W–VO_2_ is a typical thermochromic material that can adaptively change its optical properties in the infrared wavelength. Its optical constants [28] are shown in Figure 3(a and b), and the optical constants of other materials are shown in Supplementary Material 1. In the insulating state, the extinction coefficient of W–VO_2_ is relatively tiny in all relevant wavelength ranges. However, after the insulating-metallic state transition, it does not change much in the solar spectral band, whereas it grows significantly in the near-infrared band. The sharp change in before and after the phase transition indicates that W–VO_2_ has a solid thermal modulation [29].

In order to further enhance the thermal modulation of W–VO_2_, we employed a Fabry–Perot resonant cavity structure consisting of a W–VO_2_/dielectric/metal reflector similar to that described in the literature [30]. Ag was used as the substrate, POE film as the spacer layer, and a hybrid layer of POE and W–VO_2_ was used as the top layer of the Fabry–Perot resonant cavity. The reflectance spectra of Ag are shown in Figure 3(c and d), and its high reflectance in the visible and infrared wavelength bands makes it suitable to be used as the metal reflector of the F–P resonant cavity. POE has broadband transparency properties in the visible and mid-infrared wavelength bands. Its infrared transmittance is shown in Figure 3(e and f), and it can be used as a spacer material for the resonant cavity. A hybrid layer of POE and W–VO_2_ is used as the top layer of the F–P resonant cavity, which can be doped with tungsten to reduce its phase transition temperature to about room temperature (30 °C).

Meanwhile, VO_2_ is an unstable phase, and in practice, vanadium dioxide is easily converted to its oxide or hydroxide by reacting with H_2_O, O_2_, etc., in the air, thus losing its phase change properties [31], [32] To improve the stability of W–VO_2_, it is expected to reduce the contact of vanadium dioxide with oxygen and water by using a protective film. Commonly used protective film materials include silicon oxide, aluminum oxide, titanium oxide, hafnium oxide, etc. [33], [34], [35], [36]. However, conventional protective film materials have good thermal radiation capability in the 8–13 μm band, making it impossible to effectively suppress the thermal radiation capability of the radiative cooler in the low-temperature state. Therefore, it is necessary to find a new protective material. A deficient thermal radiation capacity in the 8–13 μm band is required to ensure that the radiative cooling capacity of the adaptive radiative cooler is weak enough at low temperatures. Poly(ethylene-1-octene) is highly transparent in both the visible and infrared wavelengths, and it can effectively isolate substances such as O_2_, H_2_O, etc., so we chose POE to be the antioxidation layer. In addition, the thickness of the POE and hybrid layers can be up to μm level, and the preparation is more tolerant.

**Figure 4:**
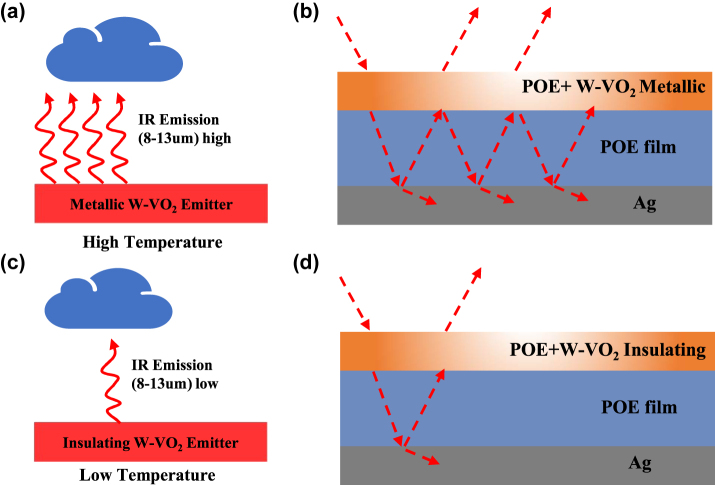
Schematic diagram of dynamic radiative cooling and its principle. (a) Infrared emission behavior of metallic W–VO_2_ at high temperature; (b) schematic diagram of metallic W–VO_2_ emitter and its optical path at high temperature; (c) infrared emission behavior of insulating W–VO_2_ at low temperature; and (d) schematic diagram of insulating W–VO_2_ emitter and its optical path at low temperature.

A hybrid layer of POE and W–VO_2_ was used as the top layer of the Fabry–Perot resonant cavity. When the temperature is higher than the phase transition point of W–VO_2_, the wave interference effect enhances the mid-infrared absorption, as shown in Figure 4(a and b), which exhibits a high infrared emissivity [37]. When the temperature is lower than the phase transition point of W–VO_2_, W–VO_2_ is insulating with a high transmittance of light in the visible and infrared bands. There is no substantial Fabry–Perot enhancement, as in Figure 4(c and d), and the emitter is in a low emissivity state, thus suppressing the radiative cooling process.

In addition to the simple structure, another advantage of the Fabry–Perot resonant cavity is the spectral tunability; the resonant wavelength and the resulting absorption enhancement can be easily tuned by changing the thickness of the cavity material [38], and the amplitude and width of the resonance response can be adjusted by the thickness of the top reflector material [39]. This tunability is especially suitable for applications such as building cooling. By rationally designing the thicknesses of the mixing and spacer layers and the ratio of W–VO_2_ in the mixing layer, the bottom radiative cooler can exhibit a high modulation rate of IR emission in the atmospheric window band.

### 2.3 Design and principle of adaptive radiative cooling system

**Figure 5:**
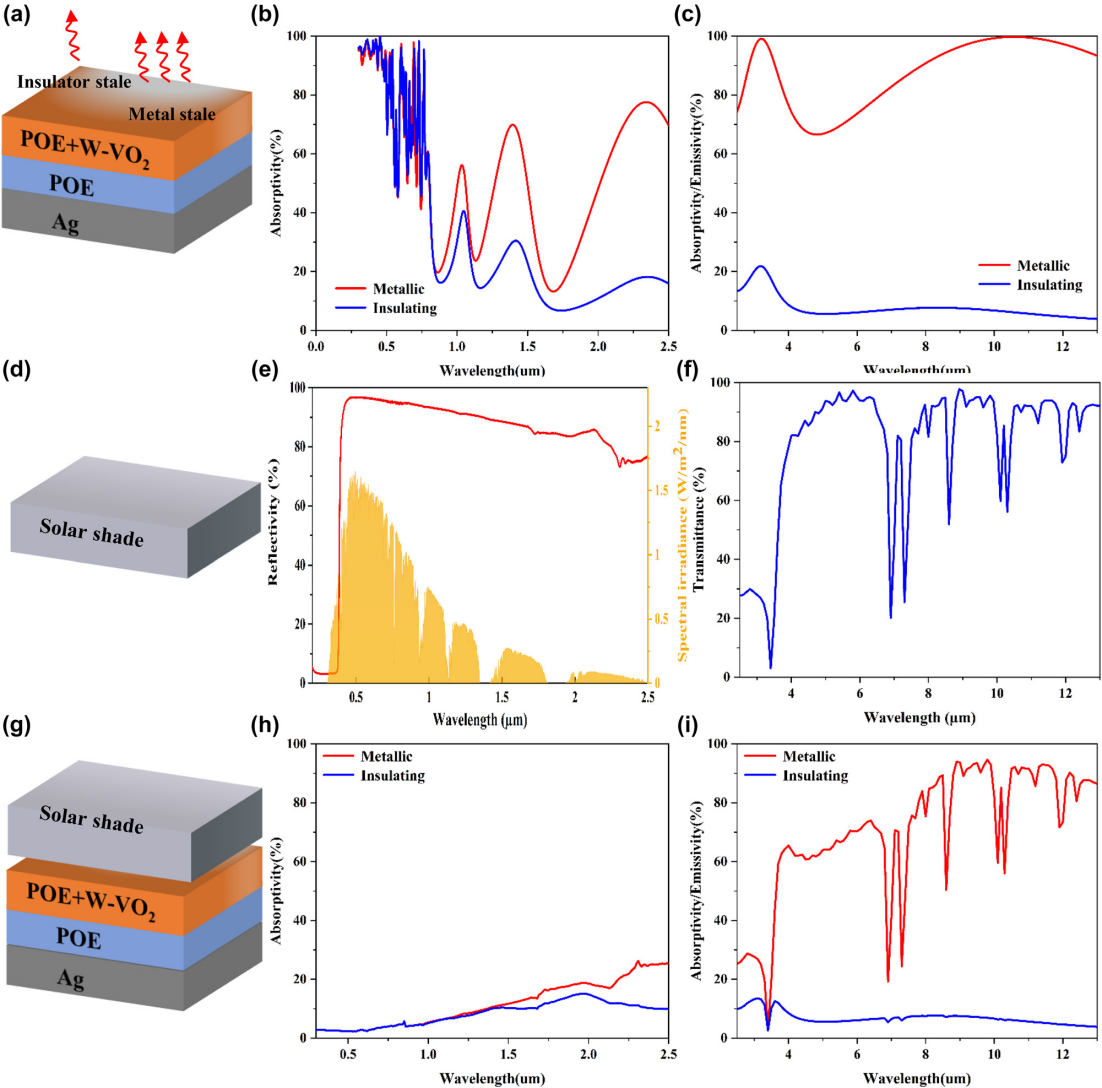
Structure and spectral diagram of the adaptive radiative cooler. (a) Schematic diagram of the lower radiative cooler; (b) absorptivity of the radiative cooler in the sunlight band; (c) emissivity of the radiative cooler in the infrared band; (d) schematic diagram of the upper solar reflector; (e) reflectivity of the solar reflector in the sunlight band; (g) schematic diagram of the combined adaptive radiative cooling system; (h) combined adaptive absorptivity of the radiative cooling system in the solar band; and (i) emissivity of the combined adaptive radiative cooling system in the infrared band.

The absorptivity and emissivity of the bottom dynamic radiative cooler in the solar and infrared bands are shown in Figure 5(b) and (c), respectively. We placed a solar reflector on top of the dynamic radiative cooler to further improve the structure’s performance for daytime radiative cooling. This solar reflector is a ZnO–PE reflector prepared by Cui’s team [40], which can achieve an average reflectivity of more than 90 % in the sunlight band and an average transmittance of about 80 % in the mid-infrared band. This solar reflector has two roles – reflecting sunlight and transmitting infrared emission. Firstly, as shown in Figure 5(e), the ZnO–PE structure prevents the sunlight from reaching the radiative cooler at the bottom due to its high reflectivity in the sunlight band. Secondly, as shown in Figure 5(f), the solar reflector has a high transmittance in the infrared wavelength range, which has little effect on the radiative heat transfer between the bottom radiative cooler and outer space.

Based on this, the structure of our designed adaptive radiative cooler is shown in Figure 5(g). The adaptive radiative cooling system consists of a solar reflector at the top and a dynamic radiative cooler at the bottom. The solar reflector at the top has high solar reflectivity to reduce the solar energy absorbed by the system. At the same time, a high infrared transmittance is maintained to allow the heat to be emitted in the form of radiative. The bottom radiative cooler is based on a W–VO_2_ phase change material that can switch between high and low emissivity depending on the temperature. For such a combined structure, we can use incoherent calculations to get the absorptivity of the combined structure, see Figure 5(h and i). Here, based on multiple reflection processes between the solar reflector at the top and the radiative cooling at the bottom, the absorptivity of the combined system is [41]:

(2-1) $$\varepsilon \left(\lambda ,\Omega \right)=T_{r}\left(1-R_{c}\right)/\left(1-R_{r}R_{c}\right)$$

where *T*_*r*_, *R*_*r*_, and *R*_*c*_ are the power transmission coefficient of the solar reflector, the power reflection coefficient of the upper surface of the radiant cooler, and the power reflection coefficient of the lower surface of the solar reflector, respectively.

## 3 Results and analysis

Based on finite element simulation simulations, we investigate the effects of single and composite factors on the adaptive radiative cooler. Focusing on the effect of the thickness of the F–P resonant cavity on the spectrum, each layer’s optimal thickness and the tolerance’s magnitude are determined.

### 3.1 Effect of volume ratio on adaptive radiative coolers

Firstly, the thickness of the POE layer in the F–P resonance cavity was set to 600 nm, and the thickness of the hybrid layer was set to 1 μm. The effects of different mixing ratios of W–VO_2_ and POE in the hybrid layer on the optical performance of the radiative cooler were explored. As shown in Supplementary Material Figure S2(a–d). When the temperature is lower than the phase transition temperature of W–VO_2_, W–VO_2_ is insulating. At this time, the absorptivity of the radiative cooler is only affected by the material itself. Therefore, the absorption in the solar band and the mid-infrared band increases with the increase of the volume share of W–VO_2_. When the temperature is higher than the W–VO_2_ phase transition temperature, W–VO_2_ is in the metallic state. At this time, the absorptivity of the radiative cooler shows a trend of increasing and then decreasing with the increase of the volume share of W–VO_2_. This is because when the amount of W–VO_2_ is insufficient, the reflectivity of the top layer is insufficient, and the incident light cannot oscillate effectively in the resonant cavity.

**Figure 6:**
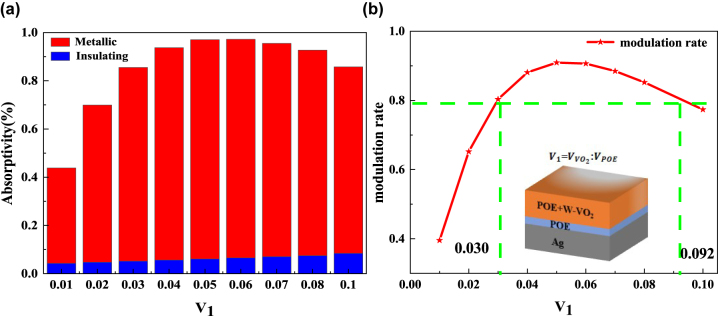
Effect of volume ratio on adaptive radiative coolers. (a) The average emissivity of the adaptive radiative cooler in the atmospheric window for different volume ratios; (b) modulation rate of the adaptive radiative cooler for different volume ratios (red line); the green line indicates the volume ratio corresponding to a modulation rate of 0.8.

Moreover, when there is too much W–VO_2_, the reflectivity is too high, and the incident light cannot enter the resonant cavity, which decreases the absorption rate. From Figure 6(a and b), it can be seen that the modulation rate of the radiative cooler in the atmospheric window (the difference between the emissivity at high and low temperatures) also shows a trend of first increasing and then decreasing with the increase of the W–VO_2_ volume share. When the W–VO_2_ volume ratio is 0.05, the modulation rate of the radiative cooler in the atmospheric window band is the highest and can reach 0.909. Meanwhile, to keep the modulation rate of the radiative cooler above 0.8, the volume ratio of W–VO_2_ can be varied between 0.030 and 0.092.

### 3.2 Effect of mixed layer thickness on adaptive radiative coolers

In addition, the thickness of the POE layer in the F–P resonant cavity was set to 600 nm, and the volume share of W–VO_2_ in the hybrid layer was set to be constant at 0.05. We explored the effect of the thickness of the hybrid layer on the optical performance of the adaptive radiative cooler, as shown in Figure S3(a–d) in Supplementary Material. When W–VO_2_ is in the metallic state, the absorptivity of the radiative cooler in the solar and mid-infrared bands shows an increasing and then decreasing trend with the thickness of the two-phase blended film. This is because the volume share of W–VO_2_ and the thickness of the two-phase hybrid film are proportional to the mass of W–VO_2_, so the influence law is the same as the previous one. When W–VO_2_ is insulating, the absorption rate changes in the same way.

**Figure 7:**
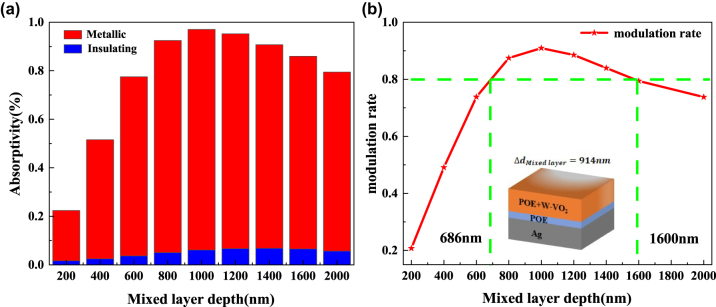
Effect of mixing layer thickness on adaptive radiative coolers. (a) Average emissivity in the atmospheric window for adaptive radiative coolers with different mixing layer thicknesses; (b) modulation rate of adaptive radiative coolers with different mixing layer thicknesses (red line); the green line indicates the corresponding mixing layer thickness for a modulation rate of 0.8.

Meanwhile, the thickness of the hybrid layer reaches the μm level, which is much thicker than the W–VO_2_ layer alone. The hybrid layer absorbs part of the light, and the spacer layer and the hybrid layer participate in the F–P resonance, increasing the absorption depth of the light resulting in a higher emissivity than the W–VO_2_ layer alone as the top layer. As shown in Figure 7(a and b), when the thickness of the hybrid layer is 1 μm, the absorptivity and modulation rate of the radiative cooler reach the maximum value in the atmospheric window, which is 0.970 and 0.909, respectively, under the condition of fixed POE layer thickness and mixing ratio. To achieve a modulation rate of 0.8 or more in the atmospheric window, the thickness of the hybrid layer can be varied between 686 nm and 1600 nm. It is further demonstrated that the hybrid layer is prepared with a large tolerance.

### 3.3 Effect of spacer thickness on adaptive radiative coolers

The volume percentage of W–VO_2_ in the hybrid layer was set to 0.05, and the thickness of the hybrid layer was kept constant at 1 μm. Supplementary Material Figure S4(a–d) shows the effect of the thickness of the spacer layer POE film on the absorptivity of the radiative cooler. The figure shows that the POE film’s thickness has no significant effect on the overall absorptivity of the radiative cooler, regardless of whether the W–VO_2_ is in the metallic or insulating state. Changing the POE film’s thickness only changes the resonance wavelength’s magnitude, while the resonance response’s amplitude and width do not change significantly. Although the peak and width of the absorptivity remain unchanged, shifting the resonance wavelength changes the absorptivity of the radiative cooler in the atmospheric window.

**Figure 8:**
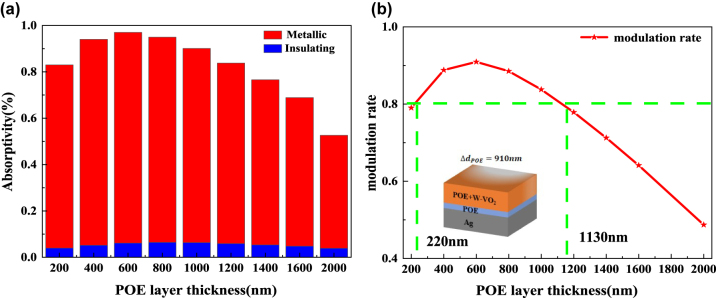
Effect of spacer thickness on adaptive radiative coolers. (a) Average emissivity in the atmospheric window for adaptive radiative coolers with different spacer thicknesses; (b) modulation rate of adaptive radiative coolers with different spacer thicknesses (red line); the green line indicates the corresponding spacer thickness at a modulation rate of 0.8.

As shown in Figure 8(a and b), the emissivity and modulation rate of the radiative cooler in the atmospheric window band tends to increase and decrease with the increase of POE film thickness. The maximum values are reached at a POE film thickness of 600 nm, which are 0.970 and 0.909, respectively. Under the fixed mixing layer thickness and mixing ratio condition, the modulation rate in the atmospheric window is necessary to be more than 0.8. The thickness of the POE layer can be varied between 220 nm and 1130 nm, which indicates that the spacer layer is also prepared with a large fault tolerance.

### 3.4 Effect of compound factors on adaptive radiative coolers

**Figure 9:**
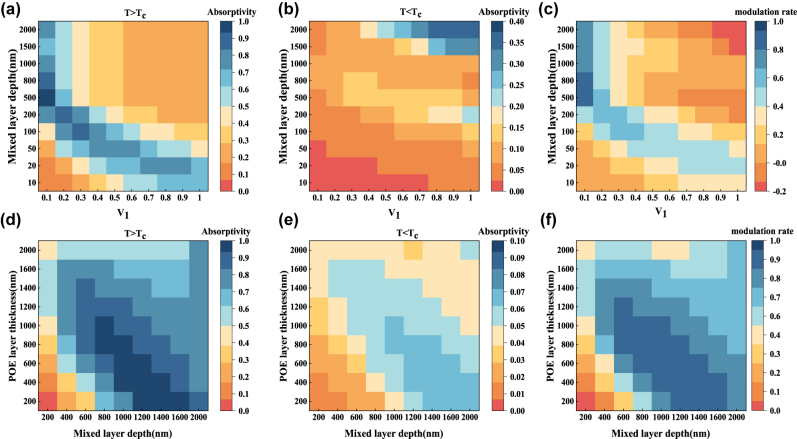
Effects of W–VO_2_ volume share, hybrid layer thickness, and POE film thickness on the optical performance of the adaptive radiative cooler. (a) Effect of W–VO_2_ volume share and hybrid layer thickness change on the absorptivity of the radiative cooler in the atmospheric window band when W–VO_2_ is in the metallic state; (b) effect of W–VO_2_ volume share and hybrid layer thickness change on the absorptivity of the radiative cooler in the atmospheric window band when W–VO_2_ is in the insulating state; (c) effect of W–VO_2_ volume share and hybrid layer thickness change on the modulation rate of the radiative cooler in the atmospheric window band; (d) the effect of mixing layer thickness and POE film thickness change on the radiative cooler’s absorptivity in the atmospheric window band when W–VO_2_ is in the metallic state; (e) the effect of mixing layer thickness and POE film thickness change on the radiative cooler’s absorptivity in the atmospheric window band when W–VO_2_ is in the insulating state; and (f) the effect of varying the thickness of the mixed layer and the POE film’s thickness on the radiative cooler’s modulation rate in the atmospheric window band.

The above investigation was done for the law of the influence of a single factor on the optical performance of an adaptive radiative cooler. In order to select the optimal volume share of W–VO_2_ and the thickness of each layer, the law of multiple factors affecting the radiative cooler is investigated. The thickness of the spacer layer POE film was kept constant at 600 nm, and the volume ratio of W–VO_2_ in the hybrid layer and the thickness of the hybrid layer were varied. The results are shown in Figure 9(a–c), where the volume fraction of W–VO_2_ and the thickness of the two-phase blended film are both proportional to the mass of W–VO_2_, and thus both have the same effect on the emissivity and modulation rate, which are both increasing and decreasing at the same time. Setting the volume ratio of W–VO_2_ in the hybrid layer at 0.05 and keeping it constant, changing the thickness of the hybrid layer and the thickness of the spacer layer of POE film, the results are shown in Figure 9(d–f), where the POE film’s thickness only changes the resonance wavelength size, and the resonance response’s amplitude and width do not change significantly. The effects of the thickness of the hybrid layer on the emissivity and modulation rate are consistent with the previous ones.

Through the above simulations, the optimal parameters of the adaptive radiative cooler are as follows: the volume ratio of W–VO_2_ in the mixing layer is 0.05, the thickness of the mixing layer is 1 μm, and the thickness of the spacer layer POE film is 600 nm; at this time, the modulation rate of the radiative cooler in the atmospheric window band is 0.909, and the average absorptivity in the metallic state of W–VO_2_ is 0.970, and that of the insulating state of W–VO_2_ is 0.061. The average emissivity at thermal equilibrium conditions was calculated according to Kirchhoff’s law, as shown in Supplementary Material 5.

### 3.5 Radiative cooling performance

Based on Supplementary Material 6, we simulated the adaptive radiative cooler’s net radiative cooling power and cooling effect before and after the phase change temperature. When the temperature is higher than the phase change temperature (typically daytime), the adaptive radiative cooling device is in a high emissivity state at this time. Setting the solar radiant power at this time to 1000 W/m^2^ and assuming that the atmosphere is completely transparent (i.e., transparency of 1), the adaptive device’s net radiative cooling power and temperature reduction are calculated for different nonradiative heat transfer conditions (*hc* = 0, 4, 8, 12, 16, 20 W m^−2^ K^−1^). During daytime, the maximum net radiative cooling power of the adaptive device can reach 69.85 W/m^2^, as shown in Figure S5(a). When, the simulated cooling effect can be up to about 3 °C. When the temperature is lower than the phase transition temperature (usually at night), the adaptive radiative cooler is in a low emissivity state, and the radiative cooling is turned off. Currently, the calculated net radiative cooling power is only 8.74 W/m^2^, about one-eighth of the cooling effect during daytime, as shown in Figure S5(b). When *h*_*c*_ = 20 W m^−2^ K^−1^, the simulated cooling effect is only about 0.4 °C.

**Figure 10:**
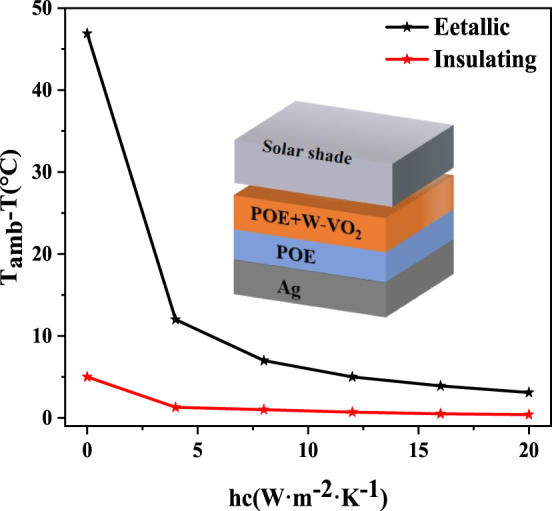
The cooling effect of the adaptive radiative cooling system under different conditions: variation of the cooling effect with convection coefficient for the adaptive radiative cooling system at the low temperature (red line) and the high temperature (black line).

Figure 10 shows the cooling effect of the adaptive radiative cooling system at high and low temperatures. The significant difference between the net radiative cooling power and the cooling situation during daytime and nighttime proves its excellent adaptive radiative cooling performance. It can realize the radiative cooling turns on or off according to the temperature change to realize the all-day dynamic thermal management.

## 4 Conclusion and outlook

In this paper, an adaptive radiative cooling system based on W–VO_2_ is designed. The system can automatically turn on the radiative cooling when the ambient temperature is higher than the critical temperature and turn off the radiative cooling when the ambient temperature is lower than the critical temperature. Using a simple Fabry–Perot resonant cavity structure, its spectra at high and low temperatures can be easily adjusted by the thickness of each layer and the ratio of W–VO_2_. The system achieves a high modulation rate of 0.909 in the atmospheric window band, and the net radiative cooling power at low and high temperatures differs by nearly eight times, demonstrating excellent adaptive performance. At the same time, the polymer film is prepared with a simple process and a high degree of fault tolerance, and it also prevents W–VO_2_ from being oxidized and losing its modulation ability. The adaptive radiative cooler has excellent dynamic thermal management capability with a simple and low-cost process. However, it is still a long way from practical application due to the cumbersome preparation process of raw VO_2_. If VO_2_ nanopowders can be prepared more straightforwardly and economically, this device will be one of the best choices for adaptive radiative cooling.

## Supplementary Material

Supplementary Material Details
