# Supplementary material for: Theoretical study of a highly fault-tolerant and scalable adaptive radiative cooler
Source: Nanophotonics. 2024 Feb 12;13(5):725–36. doi: 10.1515/nanoph-2023-0739 (PMC11501479; doi:10.1515/nanoph-2023-0739)
Supplement: Supplementary file 1 — Supplementary Material Details [file j_nanoph-2023-0739_suppl_001.pdf]

Support information

Theoretical study of a highly fault-tolerant and scalable adaptive radiative cooler

Bin Li, [a] *Jiaqi Hu,* [a] *Changhao Chen,* [a] *Hengren Hu,* [a] *Yetao Zhong,* [a] *Ruichen Song,* [a] *Yunqi Peng,* [a] *Xusheng Xia,* [a] *Kai Chen* [b] *and Zhilin Xia,*[a,*]

[a]Wuhan University of Technology, State Key Laboratory of Silicate Materials for Architectures, Wuhan, China

[b]*Wuhan Zhongyuan Huadian Science and Technology Co., Ltd., Wuhan, China*

*Corresponding author's e-mail: xiazhiiln@whut.edu.cn

Supplement 1. Refractive index n and extinction coefficient k of the material

(1) Optical constants of POE

The relative dielectric function of the medium in the presence of light absorption is[1]

$$\varepsilon(\omega) = \varepsilon_1(\omega) + i\varepsilon_2(\omega)$$

where $\varepsilon_1(\omega)$ is the real part of the relative dielectric function and $\varepsilon_2(\omega)$ is the imaginary part of the relative dielectric function.

Complex refractive index of the absorbing medium[2]:

$$\check{n}(\omega)=n(\omega) + ik(\omega)$$

where the real part $n(\omega)$ of the complex refractive index is the usually measured refractive index and the imaginary part $k(\omega)$ is the extinction coefficient.

The relationship between the complex refractive index and the complex relative dielectric function of the medium is given by:

$$[n(\omega) + ik(\omega)]^2 = \varepsilon_1(\omega) + i\varepsilon_2(\omega)$$

From the separate equality of the real and imaginary parts, we get

$$n(\omega)^2 - k(\omega)^2 = \varepsilon_1(\omega)$$

$$2n(\omega)\, k(\omega) = \varepsilon_2(\omega)$$

Using the above relations[3], through the dielectric constant and dissipation factor of POE, we obtained the refractive index n (1.48) and extinction coefficient k (0.004) of POE. Optical constants for Ag from Ref [4].

(2) Optical constants of W-VO_2

VO_2 is a typical phase transition material with its phase transition temperature near 68°C. The lattice distortion of vanadium dioxide can be fundamentally induced by a small amount of tungsten doping, which reduces its phase transition temperature and adjusts it to near room temperature[5-7]. Meanwhile, doping W hardly changes the optical properties of VO_2 before and after the phase transition. Because the amount of tungsten doping is relatively small, it has essentially no effect on the material's dielectric constant[8, 9]. Therefore, in this paper, we use the optical constants of VO_2 as an approximate substitute for the optical constants of W-VO_2 in our calculations.

The optical constants of VO_2 were calculated from the literature[10, 11] using the

same method as Fan et al[12]:

$$\varepsilon(\omega)=\varepsilon_\infty+\frac{(\varepsilon_s-\varepsilon_\infty)\cdot\omega_t^2}{\omega_t^2-\omega^2+i\Gamma_0\cdot\omega}+\sum_{j=1}^{n}\frac{f_j\cdot\omega_{oj}^2}{\omega_{oj}^2-\omega^2+i\gamma_i\cdot\omega}+\frac{\omega_p^2}{-\omega^2+i\Gamma_d\cdot\omega}$$

The specific parameters for calculating the optical constants are:

	ε_∞	ε_s	ω_t	Γ_0	ω_p	Γ_d
VO_2(M)	0.47	2.64	0.89 eV	1.21 eV	0	0
VO_2(I)	2.95	-13.90	0.86 eV	3.75 eV	4.47 eV	0.82 eV

(3) Optical constants of POE+ W-VO_2

Based on the equivalent medium theory, the effective dielectric constant of the 3D random medium is $\varepsilon_{eff}=\frac{\sum\varepsilon_i V_i}{\sum V_i}$, where V_i is the volume of the ith medium[13]. We calculate the refractive index n and the extinction coefficient k of the POE+W-VO_2 layer. The effective refractive index n of the hybrid layer is $n_{VO_2(W)}\times V_1+n_{POE}\times V_2$

The effective extinction coefficient k of the hybrid layer is $k_{VO_2(W)}\times V_1+k_{POE}\times V_2$, where V_1 and V_2 are the volume fractions of W-VO_2 and POE in the hybrid layer, respectively.

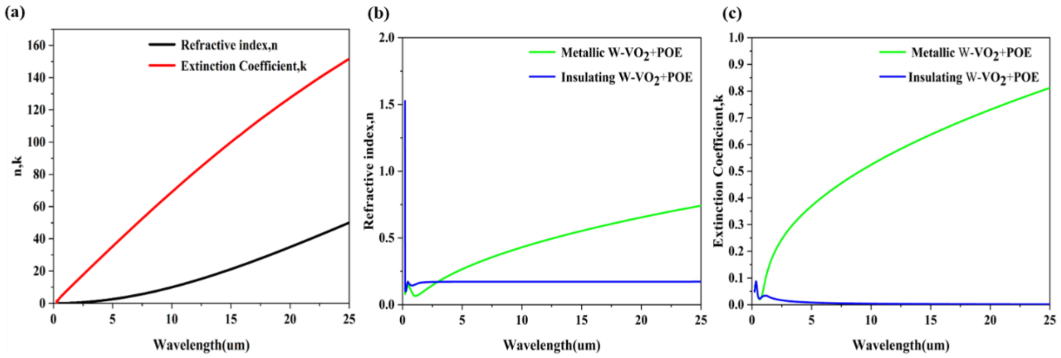

Figure S1. refractive index n and extinction coefficient k of the materials. (a) Refractive index n and extinction coefficient k of Ag; (b) refractive index n of POE+ W-VO_2 hybrid layer at high and low temperatures; (c) extinction coefficient k of POE+ W-VO_2 hybrid layer at high and low temperatures.

Supplement 2. Effect of Volume Ratio on Adaptive Radiative Coolers

The thickness of the POE layer in the F-P resonance cavity was set to 600 nm, and the thickness of the hybrid layer was set to 1 um. The effects of different mixing ratios of W-VO_2 and POE in the mixed layer on the optical performance of the radiative cooler were investigated.

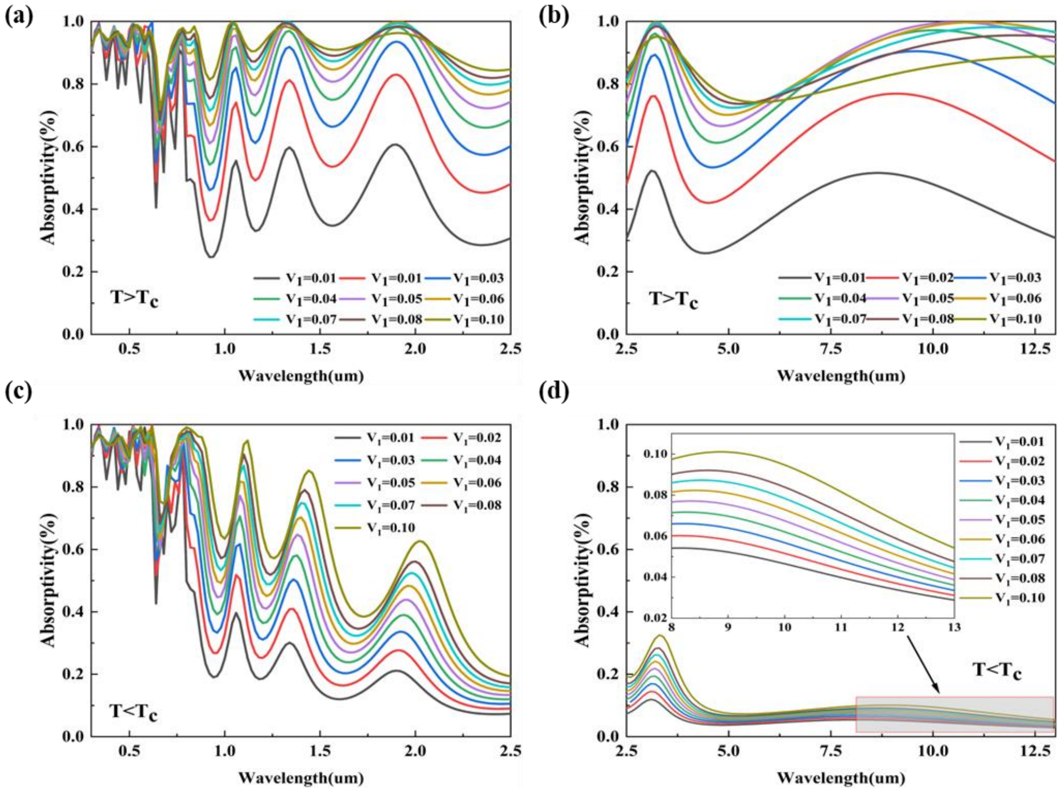

Figure S2 Effect of W-VO_2 volume occupancy on the optical performance of adaptive radiative coolers. (a) Absorbance of the radiative cooler in the visible-near-infrared (V-NIR) band when W-VO_2 is in the metallic state; (b) Absorbance of the radiative cooler in the mid-infrared (MIR) band when VO_2 is in the metallic state; (c) Absorbance of the radiative cooler in the visible-near-infrared (V-NIR) band when W-VO_2 is in the insulating state; (d) Absorbance of the radiative cooler in the mid-infrared (MIR) band when W-VO_2 is in the insulating state.

Supplement 3. Effect of Mixed Layer Thickness on Adaptive Radiant Coolers

The thickness of the POE layer in the F-P resonant cavity is set to be 600 nm, and the volume share of W-VO_2 in the hybrid layer is set to be 0.05 constant. The effect of the hybrid layer thickness on the optical performance of the adaptive radiative cooler was explored.

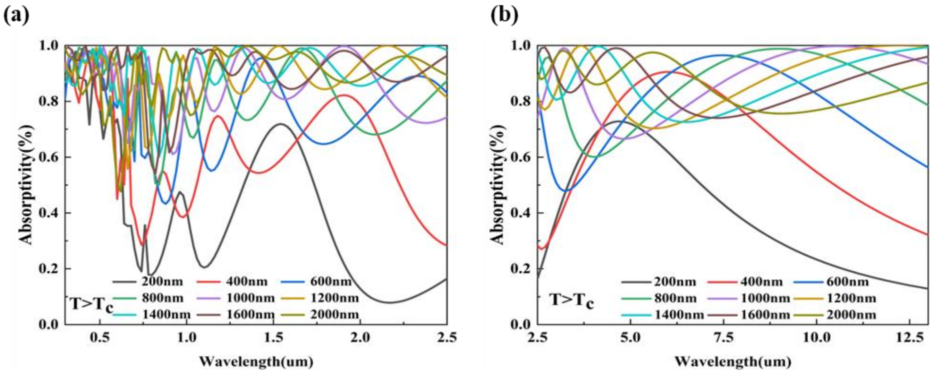

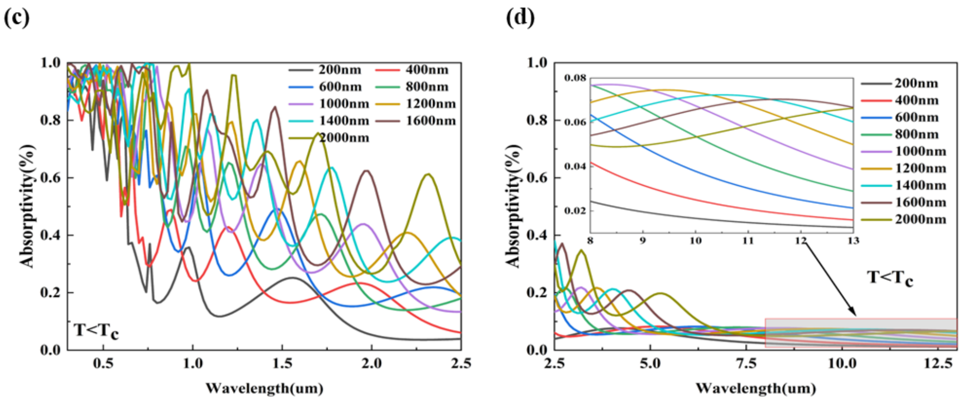

Figure S3 Effect of mixing layer thickness on the performance of adaptive radiative cooler (a) Absorption rate of the radiative cooler in the visible-near infrared (V-NIR) band when W-VO_2 is in the metallic state; (b) Absorption rate of the radiative cooler in the mid-infrared (MIR) band when W-VO_2 is in the metallic state; (c) Absorption rate of the radiative cooler in the visible-near infrared (V-NIR) band when W-VO_2 is in the insulating state; and (d) Absorption rate of the radiative cooler in the mid-infrared (MIR) band when W-VO_2 is in the insulating state.

Supplement 4. Effect of spacer thickness on adaptive radiant coolers

The volume percentage of W-VO_2 in the hybrid layer was set to 0.05, and the thickness of the hybrid layer was kept constant at 1 um. The effect of the thickness of the POE spacer layer on the optical performance of the radiant cooler was explored.

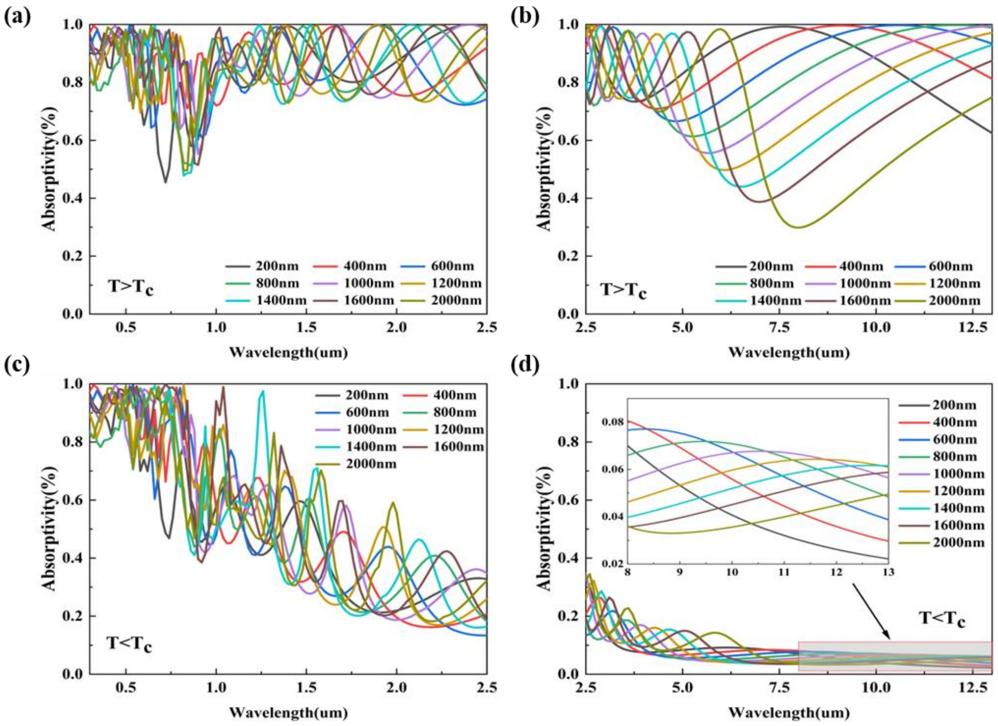

Figure S4 Effect of spacer layer POE thickness on the performance of adaptive radiative cooler (a) Absorption rate of the radiative cooler in the visible-near infrared (V-NIR) band when W-VO_2 is in the metallic state; (b) Absorption rate of the radiative cooler in the mid-infrared (MIR) band when W-VO_2 is in the metallic state; (c) Absorption rate of the radiative cooler in the visible-near infrared (V-NIR) band when W-VO_2 is in the insulating state; and (d) Absorption rate of the radiative cooler in the mid-infrared (MIR) band when W-VO_2 is in the insulating state.

Supplement 5. Average emissivity

According to Kirchhoff's law, the spectral emissivity ε is equal to the spectral absorptivity under thermal equilibrium conditions. Therefore, for opaque materials, the spectral emissivity $\varepsilon(\lambda)$ can be expressed as[14, 15]:

$$\varepsilon(\lambda) = \alpha(\lambda) = 1 - R(\lambda)$$

where $R(\lambda)$ is the spectral reflectance.

The formula is then used to integrate the spectral emissivity ε over the blackbody radiation spectrum to obtain the average emissivity ε:

$$\varepsilon = \frac{\int_{\lambda_1}^{\lambda_2}(1 - R(\lambda))B(\lambda,T)d\lambda}{\int_{\lambda_1}^{\lambda_2} B(\lambda,T)d\lambda}$$

where $B(\lambda, T)$ is the blackbody radiation at a temperature of T .

Supplement 6. Calculation of radiative cooling performance

In order to further demonstrate the effectiveness of the adaptive radiative cooler, the net radiative cooling power has been calculated at both high and low temperatures. Generally, a radiative cooler's net radiative cooling power can be defined as follows[16].

$$Q_{net} = Q_{rad} - Q_{atm} - Q_{nonrad} - Q_{sun} \quad (1)$$

Where Q_{sun} is the solar radiation power absorbed by the radiant cooler, $Q_{sun} = P_{sun} \cdot \varepsilon_1$ during the daytime, ε_1 is the solar absorption rate, and it is assumed that Q_{sun}=1000W/m^2 during the daytime and $Q_{sun} = 0$ during the nighttime.

where the radiant power Q_{rad} emitted by the radiator is

$$Q_{rad} = 2\Pi \int_0^{\frac{\Pi}{2}} sin\theta \; cos\theta \; d\theta \int_0^{\infty} U_B(T_r, \lambda) \; e_r(\lambda, \theta) d\lambda \quad (2)$$

The atmospheric radiant power Q_{atm} absorbed by the radiator is

$$Q_{atm} = 2\Pi \int_0^{\frac{\Pi}{2}} sin\theta \; cos\theta \; d\theta \int_0^{\infty} U_B(T_a, \lambda) \; e_r(\lambda, \theta) \; e_a(\lambda, \theta) d\lambda \quad (3)$$

Here, $U_B(T, \lambda) = \frac{2hc^2}{\lambda^5} \frac{1}{e^{hc/\lambda k_B T} - 1}$ is the intensity of the spectral radiation of the blackbody at temperature T, where h is Planck's constant, k_B is Boltzmann's constant, c is the speed of light in vacuum, and λ is the wavelength. According to Kirchhoff's law, the emissivity of a radiator can be determined by its absorptivity $e_r(\lambda, \theta)$, and $e_a(\lambda, \theta)$ is the atmospheric emissivity with respect to the angle. The absorptivity is determined by the angle-dependent atmospheric emissivity, $e_a(\lambda, \theta) = 1 - t(\lambda)^{1/cos\theta}$, where $t(\lambda)$ is the atmospheric transmittance in the zenith direction. T_r is the temperature of the radiator and T_a is the ambient temperature[17].

The non-radiative (conduction + convection) heat transfer from the radiator to the surroundings can be defined as

$$Q_{nonrad} = \mathrm{h}_c(T_{amb} - T_r) \quad (4)$$

Where, h_c is the sum of non-radiant heat coefficients resulting from heat transfer and convective heat exchange between the radiator and the surroundings. It is expressed as: $\mathrm{h}_c = h_{cond} + h_{conv}$. The cooling temperature is calculated with $Q_{net} = 0$.

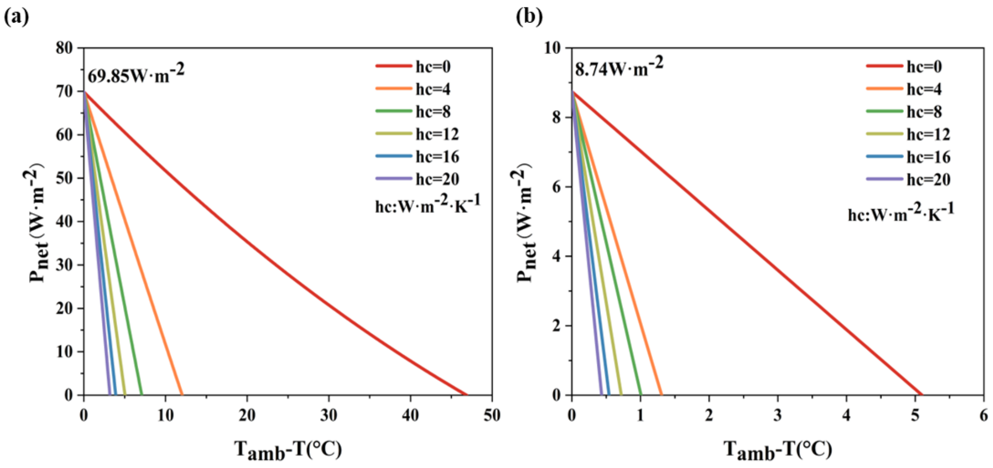

Figure S5. The cooling effect of the adaptive radiative cooling system under different conditions: (a) variation of the net radiative cooling power with convection coefficient for the adaptive device during the daytime (high temperature); (b) variation of the net radiative cooling power with convection coefficient for the adaptive device during the nighttime (low temperature).

References

1. G. Ghosh, "Dispersion-equation coefficients for the refractive index and birefringence of calcite and quartz crystals," *Optics Communications* **163** (1), 95-102 (1999).
2. G. Kresse and J. Hafner, "Ab initio molecular dynamics for liquid metals," *Physical Review B* **47** (1), 558-561 (1993).
3. H. P. Zhao et al., "Passive radiative temperature regulator: Principles and absorption-emission manipulation," *Solar Energy Materials and Solar Cells* **229**, 9 (2021).
4. H. U. Yang et al., "Optical dielectric function of silver," *Physical Review B* **91** (23), 235137 (2015).
5. R. Shi et al., "Phase management in single-crystalline vanadium dioxide beams," *Nature Communications* **12** (1), 9 (2021).
6. X. G. Tan et al., "Unraveling Metal-insulator Transition Mechanism of VO_2 Triggered by Tungsten Doping," *Sci Rep* **2**, 6 (2012).
7. J. M. Booth and P. S. Casey, "Anisotropic Structure Deformation in the VO_2 Metal-Insulator Transition," *Physical Review Letters* **103** (8), 4 (2009).
8. S. L. Dou et al., "A facile method for the preparation of W-doped VO_2 films with lowered phase transition temperature, narrowed hysteresis loops and excellent cycle stability," *Mater. Chem. Phys.* **215**, 91-98 (2018).
9. B. G. Chae and H. T. Kim, "Effects of W doping on the metal–insulator transition in vanadium dioxide film," *Physica B: Condensed Matter* **405** (2), 663-667 (2010).

10. J. B. K. Kana et al., "Thermally driven sign switch of static dielectric constant of VO_2 thin film," *Opt. Mater.* **54**, 165-169 (2016).
11. M. M. Qazilbash et al., "Infrared spectroscopy and nano-imaging of the insulator-to-metal transition in vanadium dioxide," *Physical Review B* **79** (7), 10 (2009).
12. M. Ono et al., "Self-adaptive radiative cooling based on phase change materials," *Optics Express* **26** (18), A777-A787 (2018).
13. S. R. Liang et al., "Tunable smart mid infrared thermal control emitter based on phase change material VO_2 thin film," *Applied Thermal Engineering* **232**, 8 (2023).
14. A. Hendaoui et al., "VO_2-based smart coatings with improved emittance-switching properties for an energy-efficient near room-temperature thermal control of spacecrafts," *Solar Energy Materials and Solar Cells* **117**, 494-498 (2013).
15. E. A. Goldstein, A. P. Raman and S. H. Fan, "Sub-ambient non-evaporative fluid cooling with the sky," *Nature Energy* **2** (9), 7 (2017).
16. M. M. Hossain and M. Gu, "Radiative Cooling: Principles, Progress, and Potentials," *Advanced Science* **3** (7), 10 (2016).
17. Z. B. Yang and J. Zhang, "Bioinspired Radiative Cooling Structure with Randomly Stacked Fibers for Efficient All-Day Passive Cooling," *Acs Applied Materials & Interfaces* **13** (36), 43387-43395 (2021).
